# Denoising for high-magnification bioluminescence imaging of cells by machine learning with physics-based noise modeling of EMCCD

**DOI:** 10.2142/biophysico.bppb-v23.0025

**Published:** 2026-07-25

**Authors:** Tetsuichi Wazawa, Haiyang Jiang, Ryohei Ozaki-Noma, Yinqiang Zheng, Imari Sato, Takeharu Nagai

**Affiliations:** 1 SANKEN, The University of Osaka, Ibaraki, Osaka 567-0047, Japan; 2 Transdimensional Life Imaging Division, Institute for Open and Transdisciplinary Research Initiatives, The University of Osaka, Suita, Osaka 565-0871, Japan; 3 Next Generation Artificial Intelligence Research Center, The University of Tokyo, Tokyo 113-8656, Japan; 4 Digital Content and Media Sciences Research Division, National Institute of Informatics, Tokyo 101-8430, Japan

**Keywords:** live cell imaging, green enhanced Nano-lantern, EMCCD, optical microscopy, convolutional neural network

## Abstract

Bioluminescence imaging (BLI) detects light emission from samples expressing luciferase in the presence of luciferin. Unlike fluorescence imaging, BLI does not require excitation light, thereby avoiding phototoxicity in living cells and photobleaching of labels. However, the intrinsically slow turnover of the luciferin-luciferase reaction often results in low luminescence intensity, which degrades image quality and limits practical applications, particularly in high-magnification optical microscopy of cells. In this study, we demonstrate that image denoising provides an effective strategy to overcome this fundamental limitation of BLI. We developed a physics-based noise model that incorporates fixed-pattern noise, blooming noise, readout noise, quantization noise, and Poisson shot noise, enabling accurate estimation of noise parameters encountered in EMCCD-based microscopy. In the denoising pipeline, raw noisy images were first corrected for the fixed-pattern and blooming noises, followed by restoration using Uformer neural network trained with paired ground-truth images and synthetically generated noisy images. Quantitative evaluation using the peak signal-to-noise ratio (PSNR) and structural similarity index measure (SSIM) showed that denoised images derived from raw data acquired with exposure times of ≤100 ms achieved PSNR and SSIM values comparable to those of raw images acquired with exposure times of 1–3 s. These results indicate that the proposed denoising approach substantially extends the practical limit of high-magnification BLI.

## Significance

Bioluminescence imaging (BLI) relies on luciferase-luciferin luminescence emission without excitation light, enabling microscopy observation free from phototoxicity and photobleaching. However, intrinsically low luminescence intensity often results in poor image quality, limiting high-magnification cellular imaging. Here, we demonstrate that the image denoising provides an effective solution to this challenge. We developed an EMCCD-specific noise model to generate realistic noisy images from ground truth data and used them to train a convolutional neural network. The trained network successfully reconstructs high-quality images from low-light inputs, thereby extending the practical limits of high-magnification BLI.

## Introduction

Bioluminescence imaging (BLI) is an imaging technique that exploits the enzymatic reaction between luciferase and its substrate, luciferin. Luciferase catalyzes the oxidation of luciferin to generate oxidized luciferin, which subsequently transitions to the ground state with the emission of a luminescence photon [1,2]. In BLI, luciferase is introduced into biological samples as a genetic label and observed in the presence of luciferin. Because luciferase is a protein that can be expressed as a transgene, BLI has become an important bioimaging modality, comparable to fluorescent protein-based bioimaging [3]. Unlike fluorescence imaging, BLI does not require excitation light, and is therefore free from phototoxicity and photobleaching. This property also makes BLI highly compatible with optogenetic tools. However, the catalytic turnover of luciferase is often slow (e.g., *k*_cat_≤40 s^–1^ for NanoLuc, a very bright variant of luciferase [4]), resulting in a limited rate of photon emission. Consequently, the signal-to-noise ratio (SNR) of BLI images is often low unless long exposure times are used. Optical microscopy is widely employed to visualize organelles and protein complexes in living cells. Because such intracellular structures typically have submicrometer dimensions, they are commonly observed at high magnification (e.g., 100×) using high-sensitivity cameras with pixel size of approximately 10 μm. Importantly, the irradiance at the image plane, defined as the optical power per unit area, is inversely proportional to the square of magnification. As a result, in high-magnification BLI, the irradiance at the detector can become extremely low due to the combined effects of slow luciferase turnover and high optical magnification. Under such low-irradiance conditions, bioluminescence signals are strongly affected by noises, including camera-intrinsic noise (e.g., readout noise) as well as shot noise arising from the stochastic arrival of small number of photons, which follows Poisson statistics.

Image denoising is a computational approach to restore low-noise image from noisy raw data and represents a potential solution for low-intensity imaging modalities including BLI. Classical denoising methods based on numerical filtering have been developed, including Non-Local Means (NLM) [5,6], block-matching 3D (BM3D) [7], and non-redundant interscale wavelet thresholding (PureDenoise) [8]. More recently, high-performance denoising approaches based on machine learning (ML) have been introduced and applied to bioimaging, including content-aware image restoration (CARE) [9], Noise2Noise [10,11], the extreme low-light denoising (ELD) [12], SRDTrans [13], and TriS-D [14]. In ML-based denoising, a convolutional neural network (CNN) is trained using large datasets consisting of paired noisy images and corresponding ground truth images, and the trained network is used to reconstruct images with improved SNR. A major challenge in developing such methods lies in accurately modeling the relationship between noisy observations and their ground truth counterparts and in generating sufficiently large training datasets. Although ML-based denoising has been widely applied to fluorescence bioimaging, only a limited number of studies have reported its application to BLI [15].

In this study, we describe an ML-based denoising framework for BLI using an electron multiplying charge coupled device (EMCCD) camera, which is a widely used type of camera for low-light bioimaging, including BLI. We recently developed a CNN-based denoising method incorporating a physics-based noise model of EMCCD-based fluorescence microscopy [16]. Here, we extend this approach to BLI. Using the noise model, we generated a large dataset of paired clean and noisy images based on the see-in-the-dark (SID) dataset [17] for training a CNN. The network was further optimized using a small image dataset of experimentally acquired EMCCD images. Because the training data were generated based on a physical noise model rather than object-specific image features, the resulting denoising pipeline is independent of sample geometry. We present the noise model and experimental estimation of noise parameters, and demonstrate the application of the proposed denoising approach to EMCCD-based BLI data.

## Materials and methods

### Outline of denoising procedure

In our denoising framework, we restored clean images from noisy bioluminescence images using Uformer, a CNN-based architecture [18]. A schematic overview of the data flow is shown in Figure 1. Bioluminescence images of dim cellular structures (“Dim bioluminescence of cells” in Figure 1) were acquired using an EMCCD camera (iXon Ultra DU-897U-CSO#BV, 512×512 pixels; Andor, Belfast, UK), yielding raw input images (“Bioluminescence image of cells” in Figure 1).

As a preprocessing step, we first corrected blooming noise and fixed pattern noise (FPN) using simple arithmetic operations based on measured camera parameters (“Remove blooming & FPN” in Figure 1). The corrected images were then processed by the trained Uformer network (“Denoise using Uformer network” in Figure 1) to restore clean images. To enable accurate noise correction and supervised training of the Uformer network, noise parameters specific to the EMCCD camera were experimentally determined. We captured model pattern images in a dark room (“Model patterns in dark room” in Figure 1) and analyzed them to estimate parameters associated with blooming noise, FPN, readout noise, and camera gain (“Analyze blooming, FPN, readout noise & gain” in Figure 1). Training datasets for the Uformer network were generated using a large collection of images from the See-in-the-Dark (SID) dataset [17] as well as smaller image datasets consisting of macroscopic scenes and bright bioluminescence images of cells acquired with the same EMCCD camera. Noisy images were synthesized from the clean images based on the measured noise parameters, enabling supervised training of the network under noise conditions representative of EMCCD-based bioluminescence imaging.

### Operation condition of the EMCCD

Unless otherwise specified, the Andor iXon Ultra EMCCD camera was operated under the following conditions: EM gain, 300; preamplifier gain, 3; readout rate, 17 MHz; vertical shift speed, 3.3 μs pixel^–1^; vertical clock voltage, normal; baseline clamp, enabled with an offset of 100 counts; binning, none; sensor temperature, –60°C.

### Noise model of BLI using EMCCD

We constructed a noise model to describe BLI acquired with an EMCCD camera. The model incorporates Poisson shot noise (**N**_P_), FPN (**N**_FP_), readout noise (**N**_RO_), quantization noise (**N**_q_), and blooming noise (*B*[*]). In this framework, we assumed that the digitized output image from the EMCCD, **D**, is expressed as

(1) $$D=B\left[K\times \left(I+f\times N_{P}\left(I\right)\right)\right]+N_{\text{FP}}+N_{\text{RO}}+N_{q},$$

where **I** denotes the expected number of photonelectrons generated in the EMCCD sensor due to incident photons, *B*[*] represents the blooming artifact and is defined as a function of the image prior to blooming (Table 1). The factor *f* accounts for excess noise associated with the stochastic multiplication process of the EMCCD. *K* is the overall system gain (counts per photoelectron), incorporating both the electron-multiplying gain and the analog-to-digital conversion factor. The bold symbols (**D**, **I**, and **N**) denote image data represented in matrix form. For example, (**D**)_*i*,*j*_ indicates the digitized intensity value (counts) at pixel position (*i*, *j*), where *i* and *j* correspond to the row and column numbers of the pixel, respectively. The noise components (**N**_FP_)*_i,j_* and (**N**_RO_)*_i,j_* depend on the exposure time and the pixel position (*i*, *j*), as described below. Symbols used in the noise model are summarized in Table 1.

### Fixed pattern noise (FPN)

The intensity recorded at each pixel was assumed to consist of photoelectrons accumulated at that pixel superimposed on a baseline offset, which we define as FPN. To quantify FPN, the EMCCD camera was sealed with an end cap and placed in a dark room, and 1,000 frames of dark images were acquired at various exposure times. For a given exposure time *t*_ex_, the FPN, **N**_FP_(*t*_ex_), was calculated as the pixel-wise average of the 1,000 frames. The measured FPN exhibited clear spatial dependence on pixel position (*i*, *j*) across the image frame (Figure 2A) and also showed systematic dependence on exposure time (Figure 2C). These observations indicate that both spatial and temporal factors must be taken into account for accurate FPN correction in EMCCD-based bioluminescence imaging. In addition to FPN, observations using an EMCCD may also encounter illumination-dependent fixed pattern noise referred to as photo-response non-uniformity (PRNU), which is attributable to pixel-to-pixel sensitivity variation. Although PRNU of many commercialized EMCCD cameras would typically be <1% (PRNU=0.234% for our EMCCD according to the performance sheet), the Poisson shot noise relative to a signal can be 1% if the signal is e.g. 10,000 photonelectrons, as this ratio is described by *I*^–1/2^, where *I* is a signal (photonelectrons). Therein, we note that the signal of 10,000 photoelectrons is equivalent to 109,000 counts for the EMCCD camera by using the overall gain at an EM gain of 300 (Figure 2F), which is much larger than its unsigned 16-bit pixel depth (65,535 counts) and typical signals in BLI (see **Bioluminescence images of cells**). Thus, by approximation, we neglected PRNU in the present study.

### Readout noise

The pixel intensity at each pixel even in dark images varies from frame to frame, and this variation of pixel intensities is denoted as readout noise. In the present study, we assumed that this readout noise also contributed to the uncertainty of pixel intensities in acquired images. The readout noise thereof was estimated from the same dark image datasets acquired for the FPN measurements (see above). Let **D**_Dark,*k*_(*t*_ex_) denote the *k*-th frame of the dark image dataset captured at an exposure time of *t*_ex_. The readout noise **σ**_RO_ was calculated as

(2) $${\left(\sigma_{\text{RO}}\left(t_{\text{ex}}\right)\right)}_{i,j}={\left\{\frac{1}{m-1}{\sum_{k=1}^{m}\left[{\left(D_{\text{Dark},k}\left(t_{\text{ex}}\right)\right)}_{i,j}-{\left(N_{\text{FP}}\left(t_{\text{ex}}\right)\right)}_{i,j}\right]}^{2}\right\}}^{1/2},$$

where *m* is the total number of acquired frames (*m*=1,000 in this study). A representative spatial distribution of **N**_RO_ at an exposure time of 10 ms is shown in Figure 2B. The readout noise measured in this manner would include contributions not just from electronic readout processes but also from dark current fluctuations and gain-related variability [19].

### Overall gain of EMCCD

The overall system gain of the EMCCD camera (*K* in Eq. (1)) was determined using two independent approaches: the mean-variance method [12] and the gain-series method [20]. The gain values obtained by these two methods were in close agreement (Figure 2F), indicating consistency between the measurements. In subsequent analyses, the gain value determined by the gain-series method was used.

### Blooming noise

Blooming noise is an artifact inherent to EMCCD operation and appears as vertical stripe patterns in output images (Figure 3A). In the present noise model, we assume that blooming noise arises from column-wise signal leakage, whereby the mean intensity of pixels within a column of a blooming-free image is superimposed onto each pixel in the same column. This relationship is expressed as

(3) $${\left(D_{+\text{bl,}-\text{FP}}\right)}_{i,j}={\left(B\left[D_{-\text{bl,}-\text{FP}}\right]\right)}_{i,j}={\left(D_{-\text{bl,}-\text{FP}}\right)}_{i,j}+x\cdot \frac{1}{r}\cdot \sum_{i=1}^{r}{\left(D_{-\text{bl,}-\text{FP}}\right)}_{i,j},$$

where **D**_–bl,–FP_ denotes an image corrected for both blooming noise and FPN, i.e. **D**_–bl,–FP_=*B*^–1^[**D**_raw_–**N**_FP_] (**D**_raw_ is a raw image), and **D**_+bl–FP_ denotes an image corrected for FPN but not for blooming noise such that **D**_+bl,–FP_=**D**_raw_–**N**_FP_. Here, *x* is the blooming factor and *r* is the number of rows in the image (*r*=512 in this study).

To evaluate the column-wise summation term on the right-hand side of Eq. (3), we recursively compute the sum of pixel intensities in each column as,

(4) $$\begin{array}{cc} \frac{1}{r}\cdot \sum_{i=1}^{r}\;{\left(D_{+bl,-FP}\right)}_{i,j} & =\frac{1}{r}\cdot \sum_{i=1}^{r}\;\left[{\left(D_{-bl,-FP}\right)}_{i,j}+x\cdot \frac{1}{r}\cdot \sum_{k=1}^{r}\;{\left(D_{-bl,-FP}\right)}_{k,j}\right]\\ & =\frac{1}{r}\cdot \sum_{i=1}^{r}\;{\left(D_{-bl,-FP}\right)}_{i,j}+\frac{1}{r}\cdot r\cdot x\cdot \frac{1}{r}\cdot \sum_{i=1}^{r}\;{\left(D_{-bl,-FP}\right)}_{i,j}\\ & =\left(1+x\right)\cdot \frac{1}{r}\cdot \sum_{i=1}^{r}\;{\left(D_{-bl,-FP}\right)}_{i,j}, \end{array}$$

which can be rearranged to obtain an explicit formula for blooming noise correction,

(5) $$\begin{array}{cc} {\left(D_{-bl,-FP}\right)}_{i,j} & ={\left[B^{-1}\left(D_{+bl,-FP}\right)\right]}_{i,j}={\left(D_{+bl,-FP}\right)}_{i,j}-x\cdot \frac{1}{r}\cdot \sum_{i=1}^{r}\;{\left(D_{-bl,-FP}\right)}_{i,j}\\ & ={\left(D_{+bl,-FP}\right)}_{i,j}-\frac{x}{1+x}\cdot \frac{1}{r}\cdot \sum_{i=1}^{r}\;{\left(D_{+bl,-PP}\right)}_{i,j}\\ & ={\left(D_{raw}-N_{FP}\right)}_{i,j}-\frac{x}{1+x}\cdot \frac{1}{r}\cdot \sum_{i=1}^{r}\;{\left(D_{raw}-N_{FP}\right)}_{i,j}. \end{array}$$

To estimate the blooming factor *x*, we imaged a model pattern using the EMCCD camera. A bright object of uniform intensity was projected onto region 1 of the sensor (Figure 3C). Although illumination was confined to region 1, appreciable signal was detected in region 2, whereas little signal was observed in region 3, indicating the presence of blooming noise. Mean intensities (counts per pixel) were measured for 32×32 pixel subregions in regions 2 and 3, denoted as *v*_2_ and *v*_3_, respectively, and for a vertical region spanning 32×512 pixels that included the subregion for *v*_2_, denoted as *c*_12_ (Figure 3C). The blooming factor *x* was calculated as

(6) $$x\left(t_{\text{ex}}\right)=\frac{v_{2}\left(t_{\text{ex}}\right)-v_{3}\left(t_{\text{ex}}\right)}{c_{12}\left(t_{\text{ex}}\right)-v_{2}\left(t_{\text{ex}}\right)},$$

where the explicit dependence on exposure time *t*_ex_ in *x*(*) is included, as the blooming factor was found to vary with exposure time (Figure 3D). The exposure-time dependence of *x*(*t*_ex_) was empirically fitted using a rational function,

(7) $$x\left(t_{\text{ex}}\right)=\frac{a+b\cdot t_{\text{ex}}}{c+d\cdot t_{\text{ex}}},$$

where the constants *a*, *b*, *c*, and *d* were determined by nonlinear least-squares fitting.

### Preprocessing of raw images captured by the EMCCD

To decouple FPN and blooming noise from the subsequent denoising calculation, these noise components were corrected in the raw EMCCD images prior to application of the Uformer network (Figure 1). Specifically, for a raw image acquired by the EMCCD, **D**_raw_, which contains both FPN and blooming noise, the blooming correction operator defined in Eq. (5) was applied after subtraction of FPN to obtain a corrected image, **D**_–bl,–FP_.

### Training datasets for Uformer

Three image datasets were used for training the Uformer network [18]. The first dataset was derived from the SID dataset [17], which consists of macroscopic scenes. Because the ground truth images in SID were acquired using a Sony α7S II sensor and provided as color images with a resolution of 4288×2848 pixels, they were first converted into single-channel monochrome images. From these images, 512×512 pixel patches were randomly cropped to match the image format of the EMCCD camera. Noisy images were then synthesized from the cropped images using the noise model described by Eq. (8) (see **Synthesis of noisy images**), yielding paired clean and noisy images for supervised training (Figure 4A).

The second dataset consisted of macroscopic scenes acquired directly with the EMCCD camera (Figure 4B). For each ground truth image, 10 frames were captured without EM gain at exposure times ranging from 0.5 s to 8.0 s, depending on scene brightness, and averaged to reduce noise. Corresponding noisy images were acquired with an EM gain of 300 at exposure times between 1 ms and 0.1 s without frame averaging. These image pairs were used for the fine-tuning of the Uformer network.

The third dataset was derived from bioluminescence microscopy observations acquired with the EMCCD camera. For each imaging condition, 100 consecutive frames were captured at an exposure time of 3 s during observations of cytoplasm, endoplasmic reticulum, mitochondria, and nucleus, yielding 24 image datasets. The 100 frames were averaged, and the resulting images were corrected for FPN and blooming noise (Eq. (5)). Residual noise was further reduced using a median filter, and the processed images were used as ground truth. Noisy images were then synthesized from these ground truth images using Eq. (8) (see **Synthesis of noisy images**) (Figure 4C). The resulting image pairs were used for additional fine-tuning of the Uformer network.

### Synthesis of noisy images

For supervised training of the Uformer neural network (see below), noisy images were synthesized from clean images using the noise model described above. Given a clean image **S**, a corresponding noisy image **D**′ was generated by adding noise components using random number generators as

(8) $$D^{\prime }=K\left(I+f\times N_{p}\right)+N_{\text{RO}}+N_{q},$$

where the noise synthesis process is defined by Eqs. (9)–(13):

(9) $$I=\frac{S}{K},$$

(10) $$f∼\text{P}{\text{D}}_{\text{Uniform}}\left(1.3,\sqrt{2}\right),$$

(11) $${\left(I+N_{P}\right)}_{i,j}∼\text{P}{\text{D}}_{\text{Poisson}}(\left(I{)}_{i,j}\right),$$

(12) $${\left(N_{\text{RO}}\right)}_{i,j}∼\text{P}{\text{D}}_{\text{Normal}}\left(0,{\left(\sigma_{\text{RO}}\right)}_{i,j}\right),$$

and

(13) $${\left(N_{q}\right)}_{i,j}∼\text{P}{\text{D}}_{\text{Uniform}}\left(-\frac{q}{2},\frac{q}{2}\right).$$

In these equations, PD_Uniform_(*a*, *b*) denotes a uniform probability distribution between *a* and *b*, PD_Poisson_(*λ*) denotes a Poisson distribution with mean *λ*, and PD_Normal_(*μ*, *σ*) denotes a normal distribution with mean *μ* and standard deviation *σ*. **σ**_RO_ was calculated by Eq. (2) from dark image datasets that we acquired across multiple exposure times. The parameter *f* is an excess noise factor, the value of which was randomly sampled from a conventionally used range for EMCCD [19,21]. The constant *q* represents quantization step for the final digital number in the analogue to digital conversion. These probability distributions were used to model variability in camera gain, photon arrival, and electronic noise, respectively, in EMCCD-based imaging. Representative examples of synthesized noisy images generated using this procedure are shown in Figure 4.

### Training of Uformer

All training experiments were conducted using the same set of hyperparameters. Network optimization was performed using the AdamW optimizer [22] with an initial learning rate of 2×10^–4^ and a warm-up phase of 3 epochs. The batch size was set to 4, the training patch size to 512×512 pixels, and the total number of training epochs to 1,000. All remaining Uformer parameters were kept at their default values. Training was performed using two NVIDIA RTX 3090 GPUs, and each training run required approximately 25 h to complete.

### Quantification of image quality

Image quality was quantitatively evaluated using the structural similarity index measure (SSIM) and the peak signal-to-noise ratio (PSNR). Both metrics require a test image and a reference image. However, in our experiments, the dynamic ranges of pixel intensities often differed substantially between bioluminescence images and the corresponding reference images. To enable fair comparison, pixel intensities of a test image were normalized using the corresponding reference image, and then, the normalized image was applied to the calculations of SSIM and PSNR. Let **D**_R_, **D**, and **D**_N_ represent, respectively, a reference image, a test image, and a normalized image calculated from the test image **D**, which are in a matrix form with the dimension of *L*×*M* (*L* and *M* are the numbers of rows and columns, respectively; *L*=*M*=512 in this study). Specifically, we used a fluorescence image (see **Denoising of microscopy images**) and averaged long-exposure bioluminescence images (see **Comparison with other denoising techniques**) for **D**_R_. We then calculated a normalized image **D**_N_ by

(14) $$D_{N}=\alpha D+\beta U,$$

and

(15) $$\left\{\alpha ,\beta \right\}=\underset{\alpha ,\beta \in R}{argmin}{∥\left(\alpha D+\beta U\right)-D_{R}∥}_{2},$$

where **U** is a matrix with dimension *L*×*M* such that every entry is 1, *α* and *β* are scalar variables, and ||*||_2_ is the L_2_-norm.

Accordingly, for a given test image **D**, we calculated SSIM and PSNR of **D**_N_ with reference to **D**_R_.

### Cell preparation

HeLa cells were cultured in Dulbecco’s modified Eagle’s medium (DMEM, low glucose; 041-29775, Fujifilm Wako Pure Chemical Corporation, Osaka, Japan) supplemented with 10% (v/v) fetal bovine serum (FBS). Cells were seeded onto 35 mm glass-bottom dishes and transfected with a plasmid (for GeNL expression in cytoplasm, actin filaments, or nucleus [23]; the plasmid for expression in cytoplasm is available at Addgene: GeNL/pcDNA3, #85200) using polyethylenimine (PEI Max 40K; Polysciences, Warrington, PA, USA) according to the manufacturer’s instructions. Following transfection, cells were maintained in DMEM containing 10% (v/v) FBS at 37°C under 5% CO_2_ for 40–60 h. Immediately prior to microscopy observation, the culture medium was removed, and the cells were gently rinsed with 2 mL of phenol red–free DMEM/F12 medium containing L-glutamine and 2-[4-(2-hydroxyethyl)-1-piperazinyl]ethanesulfonic acid (HEPES) (11039021; Thermo Fisher Scientific). The dish was then replenished with 2 mL of the same medium for imaging.

### Wide-field fluorescence microscopy observation

HeLa cells were observed using an inverted fluorescence microscope (ECLIPSE TiE; Nikon, Tokyo, Japan) equipped with either a 100× or 60× oil-immersion objective lens (CFI Plan Apochromat Lambda D 100× Oil or 60× Oil; Nikon), an LED light source (LightEngine Spectra X; Beaverton, OR, USA), and an EMCCD camera (iXon Ultra; Andor). For fluorescence imaging, excitation light at 488±3 nm was provided by the LED light source, and images were acquired using a filter cube containing a dichroic mirror (FF495-Di03) and an emission filter (BrightLine 520/35; Semrock, Rochester, NY, USA). For BLI, Nano-Glo Luciferase Assay Substrate (Promega, Madison, WI, USA) was added to the culture medium at a final dilution of 1:500 (v/v). Images were acquired without the excitation light and without the filter cube. Microscope operation, illumination control, and image acquisition were performed using MetaMorph software (Molecular Devices, San Jose, CA, USA). Bioluminescence images were acquired at exposure times of 10, 30, 100, 300, 1,000, and 3,000 ms to capture signals across a range of intensities.

## Results and discussion

### Bioluminescence images of cells

We observed HeLa cells expressing green enhanced Nano-lantern (GeNL) [23] in cytoplasm using an inverted fluorescence microscope. Because GeNL consists of a luciferase, NanoLuc [24], and a fluorescent protein, mNeonGreen, the same cells could be observed by both BLI and fluorescence imaging. Using fluorescence imaging, we readily obtained clear images of GeNL-expressing HeLa cells with an EMCCD camera at exposure times as short as 10–30 ms under 488 nm excitation for mNeonGreen (column of “Fluorescence” in Figure 5).

In contrast, when the excitation light was turned off and imaging was switched to BLI in the same field of view, we observed that the bioluminescence signal from GeNL in the same cells was 1–2 orders of magnitude lower than the fluorescence signal. As a result, exposure times longer than 1,000 ms were required to acquire images with comparable overall intensity (columns of “Bioluminescence” in Figure 5). At shorter exposure times (30–100 ms), the bioluminescence images were severely degraded, reflecting not only camera-originated noise but also noise arising from the intrinsically low photon flux of bioluminescence signal (Figure 5). It should be noted that although the bioluminescence emission band of NanoLuc with its substrate furimazine is at 460 nm [24], the main bioluminescence emission band of GeNL is at a longer wavelength of 520 nm due to bioluminescence resonance energy transfer [23], and we observed the whole wavelength range of GeNL bioluminescence without a bandpass filter. These observations motivated the development of an image denoising approach (Figure 1) to suppress dominant noise components under low-light conditions and to enable clearer visualization of dim cellular structures in BLI. In addition, we note that the intracellular intensity distributions observed by bioluminescence differed slightly from those observed by fluorescence, even at long exposure times (e.g., *t*_ex_=3,000 ms; Figure 5). This difference likely reflects distinct intracellular penetration and distribution of luciferin compared with excitation light, rather than artifacts arising from the denoising procedure.

### Camera parameters and noise characteristics

To enable effective denoising of bioluminescence images, we measured key parameters of the EMCCD camera, including FPN, readout noise, overall gain, and blooming noise, and evaluated their relevance under typical BLI conditions. FPN represents a spatially varying baseline offset onto which the detected signal is superimposed. In our measurements, FPN was clearly non-uniform across the image frame and depended on exposure time (Figure 2A, C). This exposure-time dependence indicates that accurate FPN correction requires calibration at the same exposure time used for image acquisition; neglecting this dependence would introduce systematic bias in low-light BLI data. In contrast, readout noise exhibited substantially higher spatial uniformity across the sensor (Figure 2B) while also showing exposure-time dependence (Figure 2D). The increase in readout noise at exposure times longer than 200 ms is most likely attributable to increased dark current accumulation in the EMCCD sensor. Importantly, the readout noise closely followed a normal distribution as confirmed by a normal distribution plot (Figure 2E), and thus, the superposition of contributions from electronic readout processes, dark current fluctuations, and gain-related variability (see **Readout noise**) was collectively approximated as a Gaussian noise component for subsequent modeling and learning. Consistency between the overall gain values obtained using independent measurement methods (the mean-variance method [12] and the gain-series method [20]) (Figure 2F) further supports the reliability of the camera parameter estimation. Blooming noise manifested as vertical stripe artifacts, a characteristic feature of EMCCD imaging under certain conditions (Figure 3A). Nevertheless, by incorporating a blooming correction based on the measured blooming factor and inverse transformation *B*^–1^[*], we were able to suppress blooming artifacts, although small residual signals occasionally remained (Figure 3B). This suggests that while blooming correction improves image quality, its impact is secondary compared with correction of FPN and readout noise under typical BLI conditions. Taken together, these observations highlight that the dominant noise sources limiting high-magnification BLI are exposure time-dependent FPN, Gaussian-distributed readout noise, and Poisson shot noise arising from low photon counts, rather than blooming artifacts. This noise structure justifies the use of a physics-based noise model that explicitly incorporates these components and provides a solid foundation for subsequent ML-based denoising. Importantly, the relative contributions of individual noise sources depend on imaging conditions, underscoring the need for camera-specific and exposure-time–matched calibration when applying denoising approaches to quantitative BLI.

### Denoising of microscopy images

After correcting FPN and blooming noise in the raw EMCCD images, we applied a Uformer-based denoising procedure to restore clean bioluminescence images. Figure 6A presents representative raw and denoised images of mitochondria labeled with GeNL in HeLa cells, acquired at exposure times of 10, 30, 100, 300, 1,000, and 3,000 ms. The denoising computation required 96 ms per image for a 512×512 pixel input using the trained Uformer network, indicating that the method is compatible with near-real-time post-processing. As expected, the visibility of mitochondrial structures increased with longer exposure times in both raw and denoised images. However, at every exposure time tested, mitochondrial patterns were more clearly discernible in the denoised images than in the corresponding raw images. This qualitative improvement suggests that denoising effectively suppresses noise components that obscure fine subcellular structures under low-photon conditions. To quantitatively evaluate denoising performance, we used fluorescence images of the same field of view as reference data (Figure 6A) and computed the PSNR and SSIM. Because absolute intensity levels differed between bioluminescence and fluorescence images, each bioluminescence image was linearly adjusted by gain and offset to minimize its intensity difference from the reference fluorescence image prior to metric calculation (see **Quantification of image quality**). The resulting PSNR and SSIM values are summarized in Figure 6B and Figure 6C, respectively. Both PSNR and SSIM were consistently higher for denoised images than for raw images at all exposure times examined, confirming that the denoising procedure improves image fidelity in a quantitative manner. Notably, the results indicate that denoising can substantially reduce the exposure time required to achieve a given image quality. For example, a PSNR of 37.8 dB and an SSIM of 0.91 obtained from a raw bioluminescence image acquired at an exposure time of 1,000 ms could be achieved by denoising an image acquired at an exposure time of approximately 40 ms (Figure 6B, C). This reduction in required exposure time represents a key advantage of the present denoising approach, particularly for high-magnification BLI where photon flux is intrinsically limited. This feature of the present denoising should be useful for BLI requiring high temporal resolution such as observations of intracellular Ca^2+^ wave [23], mitochondrial fission and fusion [25], and microtubule plus-end growth [26], in which observation with long exposure times is not suitable.

In the present denoising procedure, we performed the correction of FPN and blooming noise (Eq 5) followed by the reduction of the Poisson shot noise, readout noise, and quantization noise by using the Uformer network. Therein, we assumed that the Uformer network can effectively handle these noises. In fact, we supervised the Uformer network using the training image dataset synthesized by the physics-based noise model in which FPN and blooming noise were corrected but the Poisson shot noise, readout noise, and quantization noise remained. Accordingly, the result in Figure 6 would be consistent with that assumption. Additionally, although a denoised image derived from a raw image captured at an exposure time of 10 ms also indicated improved visibility of mitochondria, the structural details in the denoised image were observed to be fairly distorted (Figure 6A). The quantiles of pixel intensities at a 90% probability in the raw images of 10, 30, and 100 ms exposure times were 23, 32, and 72 counts, respectively, corresponding to 2.1, 2.9, and 6.6 photoelectrons, respectively, according to the overall gain (Figure 2F). This may suggest that pixel intensities of an object of interest need to be at least several-fold higher than the readout noise level (17 counts; Figure 2D) and a single photoelectron level (10.9 counts; Figure 2F) for reliable denoising.

### Comparison with other denoising techniques

We compared the performance of the present denoising approach with that of representative existing denoising techniques. For this analysis, bioluminescence images were acquired from HeLa cells expressing GeNL in the cytoplasm, as well as GeNL fused to β-actin or histone 2B, at exposure times of 0.3 s or 1 s and 3 s in the same field of view (designated as “cytoplasm,” “actin,” and “H2B,” respectively, in Figure 7A). Single-frame images of H2B and cytoplasm acquired at an exposure time of 0.3 s and that of β-actin at an exposure time of 1 s were used as the raw inputs for denoising (“Raw images” in Figure 7A). Because actin filaments were visible in single denoised images derived from raw images captured at exposure times of 1 s or 3 s (See “This study” in Figure 7A), we chose an image frame with 1 s exposure time for β-actin to examine denoising methods. As reference images for quantitative evaluation, we used averaged bioluminescence images generated from 4–5 frames acquired at an exposure time of 3 s (“Average images” in Figure 7A), which served as approximate ground truth for PSNR and SSIM analysis. Application of the present denoising method resulted in substantial improvements in image quality across all three cellular structures (“This study” in Figure 7A). Quantitatively, PSNR increased by 7.40, 12.0, and 11.2 dB for actin, H2B, and cytoplasmic images, respectively, while SSIM increased by 1.7-, 1.3-, and 1.3-fold, respectively, for the corresponding structures (Figure 7B). These results indicate that the denoising performance of the proposed method is robust across distinct intracellular localizations and spatial patterns. We further applied several established denoising methods to the same raw bioluminescence images, including BM3D [7], extreme low-light denoising (ELD) [12], SRDTrans [13], and a theoretical noise model-based approach [20] (Figure 7A). Among these methods, ELD exhibited the strongest performance and achieved PSNR and SSIM values comparable to those obtained with the present approach (Figure 7B). Nevertheless, PSNR and SSIM values obtained with the present method were consistently slightly higher, and the pattern of actin filaments was more clearly visualized by the present method than by ELD. In addition, visually noticeable unnatural patterns were observed in some ELD-denoised images, particularly in actin-labeled structures (e.g. dashed circles in Figure 7A). Taken together, these comparisons demonstrate that the proposed denoising method achieves image restoration performance comparable to or exceeding that of existing state-of-the-art techniques, while maintaining structural fidelity in bioluminescence images of diverse intracellular targets.

The present denoising method involved the arithmetic correction of FPN and the blooming noise and subsequent noise reduction for the Poisson shot noise, the readout noise, and the quantization noise with the neural network based upon the physics-based noise model tailored to BLI with EMCCD. In contrast, the arithmetic correction of FPN and the blooming noise was not included in the other methods. BM3D is a nonlinear nonlocal adaptive denoising algorithm. SRDTrans used the lightweight spatiotemporal transformer network based upon the Poisson-Gaussian noise, whereas the theoretical denoising used a network based upon the Poisson-gamma-normal noise model. ELD also involved a physics-based noise model including the Poisson shot noise, readout noise, quantization noise, but the noise model was optimized for CMOS cameras. Thus, the present denoising method would be more optimized for BLI using EMCCD than the others.

### Fidelity of image intensity

Not just the visibility of objects, PSNR, and SSIM, we also evaluated how accurately the pixel intensity was preserved in the denoised images. To analyze pixel intensities in raw and denoised images, we randomly generated a set of regions of interest (ROIs) (region size, 10×10 pixels; Figure 8A) that contained bioluminescent objects in the averaged images. We then calculated a pair of mean pixel intensities of the ROIs at the same position in the raw image acquired at a given exposure time and in the corresponding denoised image. In this procedure, we calculated 50 pairs of mean pixel intensity from 50 ROIs in a pair of raw and corresponding denoised image frames, and furthermore, performed this calculation for image frames acquired at exposure times of 10–3,000 ms (4 or 5 frames for each exposure time) to cover various bioluminescence intensities. Thereby, we show a plot of pixel intensity from the denoised pixel intensity against the raw pixel intensity from images of mitochondria, actin, histone 2B, and cytoplasm (Figure 8B). The data showed that the pixel intensity from denoised images highly correlated with the pixel intensity from raw images (*R*^2^=0.998), and the denoised pixel intensity above 1,000 counts deviated from the diagonal line by as small as 2%, indicating a highly linear correlation between intensities in raw and denoised images. Additionally, denoised pixel intensities below 30 counts were somewhat scattered and tended to be slightly biased upward from the diagonal line (Figure 8B inset). The overall gain of the EMCCD camera was 10.9 counts/photoelectrons (Figure 2F), and the pixel intensity of 30 counts corresponds to as low as 2.8 photoelectrons. Accordingly, the present denoising approach offers satisfactory fidelity of pixel intensity above pixel intensities of several photoelectrons, which is likely to be the lowest boundary of pixel intensity allowing of quantitative image analysis of denoised images. Additionally, we note that this threshold of 30 counts should be near to the criterion for reliable denoising based upon the readout noise and the number of photoelectrons that we proposed in the section of **Denoising of microscopy images**, although the threshold value may need to be higher than this criterion if the background signal is also present.

## Conclusion

We developed a denoising framework that combines correction of fixed-pattern noise and blooming noise with noise reduction using a Uformer neural network. Both the noise correction procedures and the training data generation were based on experimentally measured noise parameters of the EMCCD camera, allowing realistic synthesis of noisy images for supervised learning. As a result, the proposed denoising approach is fundamentally independent of the shapes or spatial patterns of the observed samples, in contrast to content-dependent denoising methods such as CARE [9]. The denoising performance of the present method surpassed that of several representative existing techniques and effectively preserved fine cellular structures in low-light images. Furthermore, the present method performed denoising with excellent fidelity of pixel intensity. By substantially improving image quality under photon-limited conditions, this approach extends the practical limits of low-light, high-magnification microscopy, including bioluminescence imaging of living cells. The proposed denoising framework therefore provides a general strategy for expanding the applicability of BLI and other low-intensity imaging modalities in biological research.

## Conflict of interest

The data presented in this paper are available from the corresponding author upon reasonable request.

## Author contributions

Tetsuichi Wazawa: Conceptualization (equal), investigation (equal), writing–original draft (lead), writing–review & editing (equal), visualization (lead). Haiyang Jiang: Methodology (equal), software (equal), investigation (equal), visualization (supporting). Ryohei Ozaki-Noma: Investigation (supporting), writing–review & editing (supporting). Yinqiang Zheng: Conceptualization (equal), methodology (equal), software (equal), investigation (equal), writing–original draft (supporting), visualization (supporting), writing–review & editing, supervision (equal). Imari Sato: Conceptualization (equal), writing–review & editing (equal), supervision (equal). Takeharu Nagai: Conceptualization (equal), writing–review & editing (equal), supervision (equal).

## Data availability

The evidence data generated and/or analyzed during the current study are available from the corresponding author on reasonable request.

## Figures and Tables

**Figure 1:**
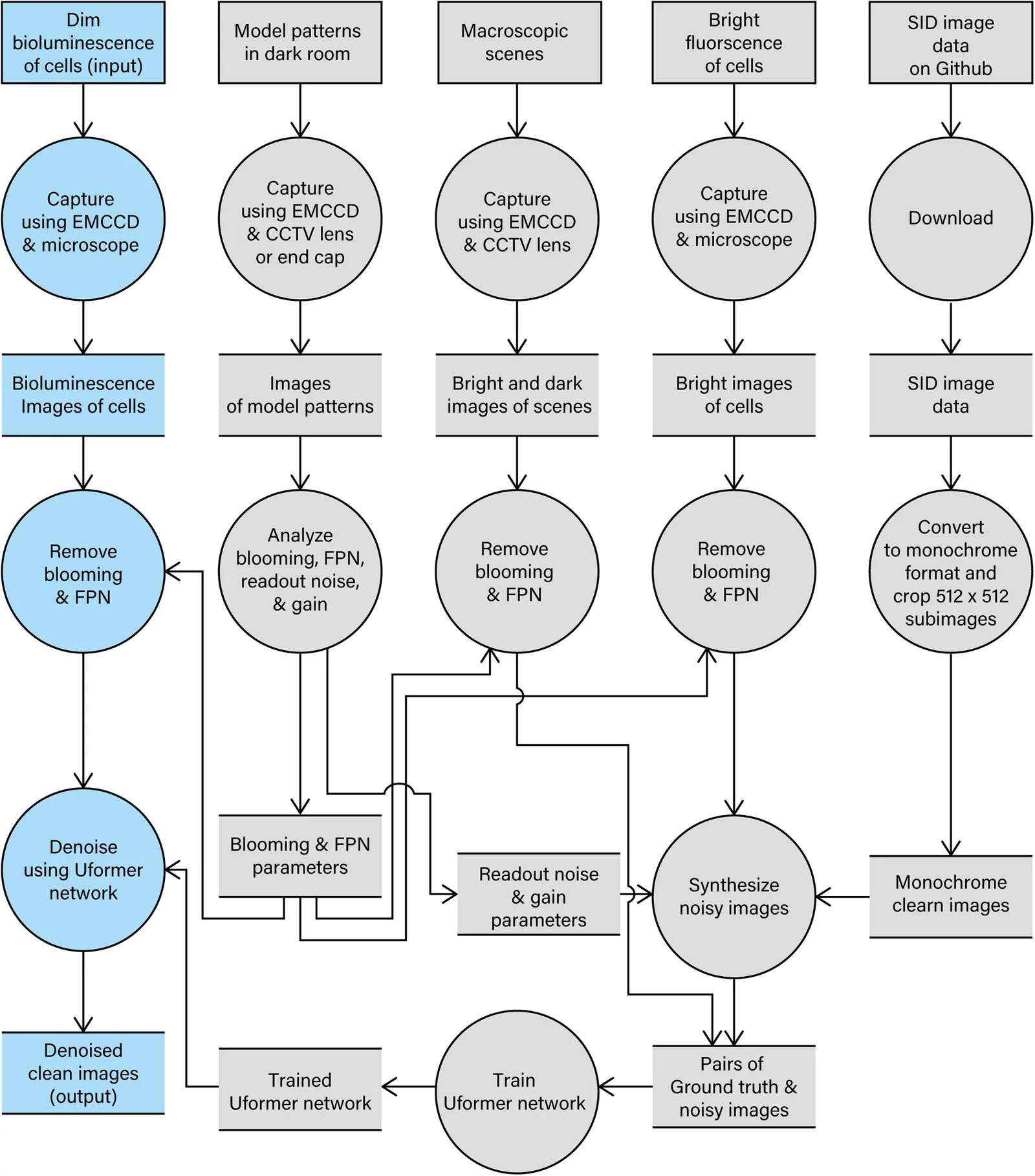
A data flow diagram (DFD) of the present denoising procedure. The blue-colored column is the main flow of operation in the present denoising. The gray colored operations are for the measurement of camera parameters, the synthesis of training data, and the training of Uformer network. The DFD is composed of external entity, data store, process, and data flow.

**Figure 2:**
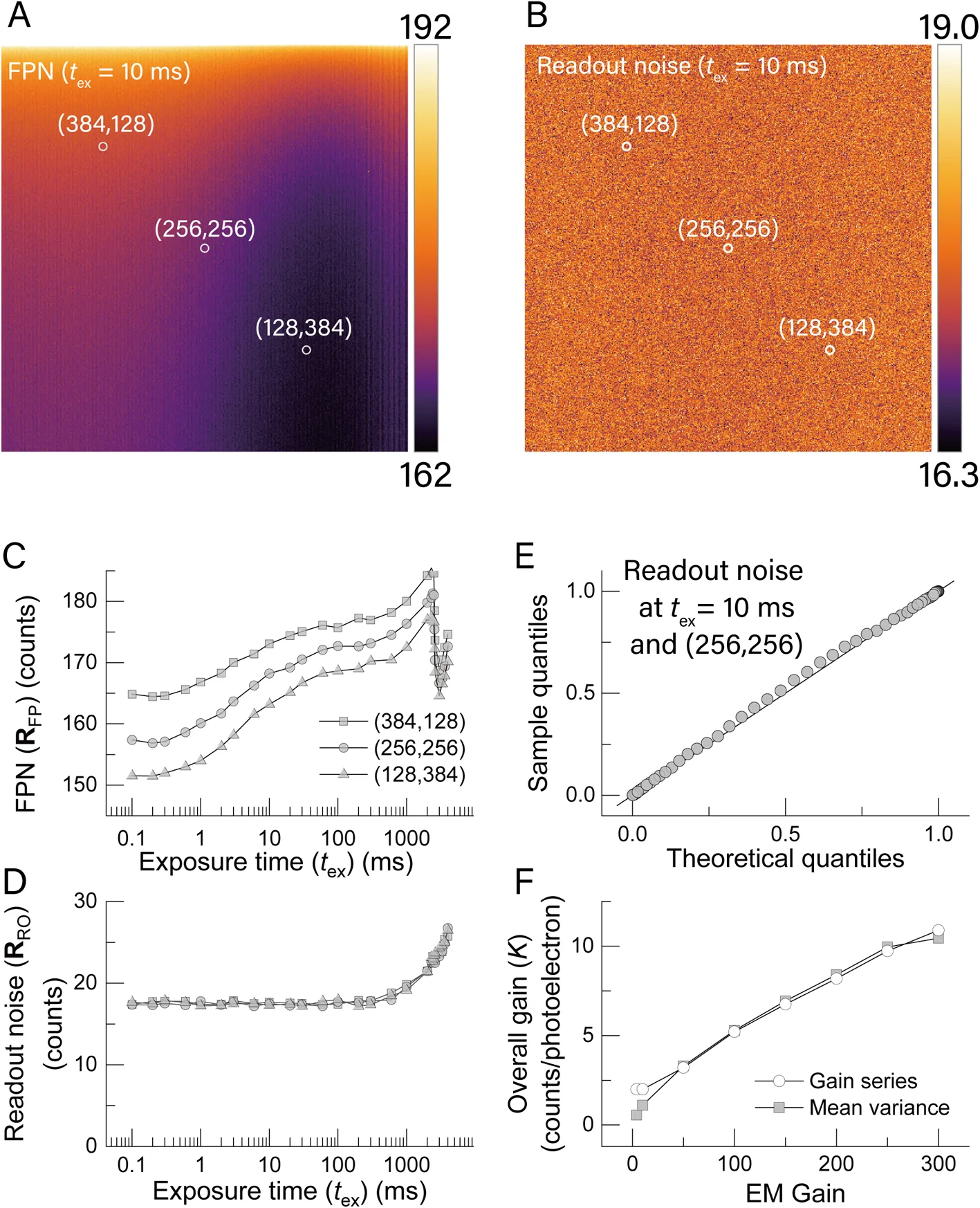
FPN, readout noise, and overall gain of the EMCCD. (A) FPN of the EMCCD in the dark at an exposure time of 10 ms. (B) The readout noise of the EMCCD in the dark at an exposure time of 10 ms as calculated by Eq. (2). (C) Pixel values of FPN at positions indicated in panel (A) at various exposure times. The values of (*,*) in panels (A) and (B) are coordinates in the image. (D) The readout noise values at positions indicated in panel (B) at various exposure times. (E) A normal probability plot of readout noise at position (256, 256) and exposure time of 10 ms. A diagonal line is also shown in this panel. (F) A plot of measured overall gain of the EMCCD by the gain series and mean variance methods as a function of user-specified EM gain.

**Figure 3:**
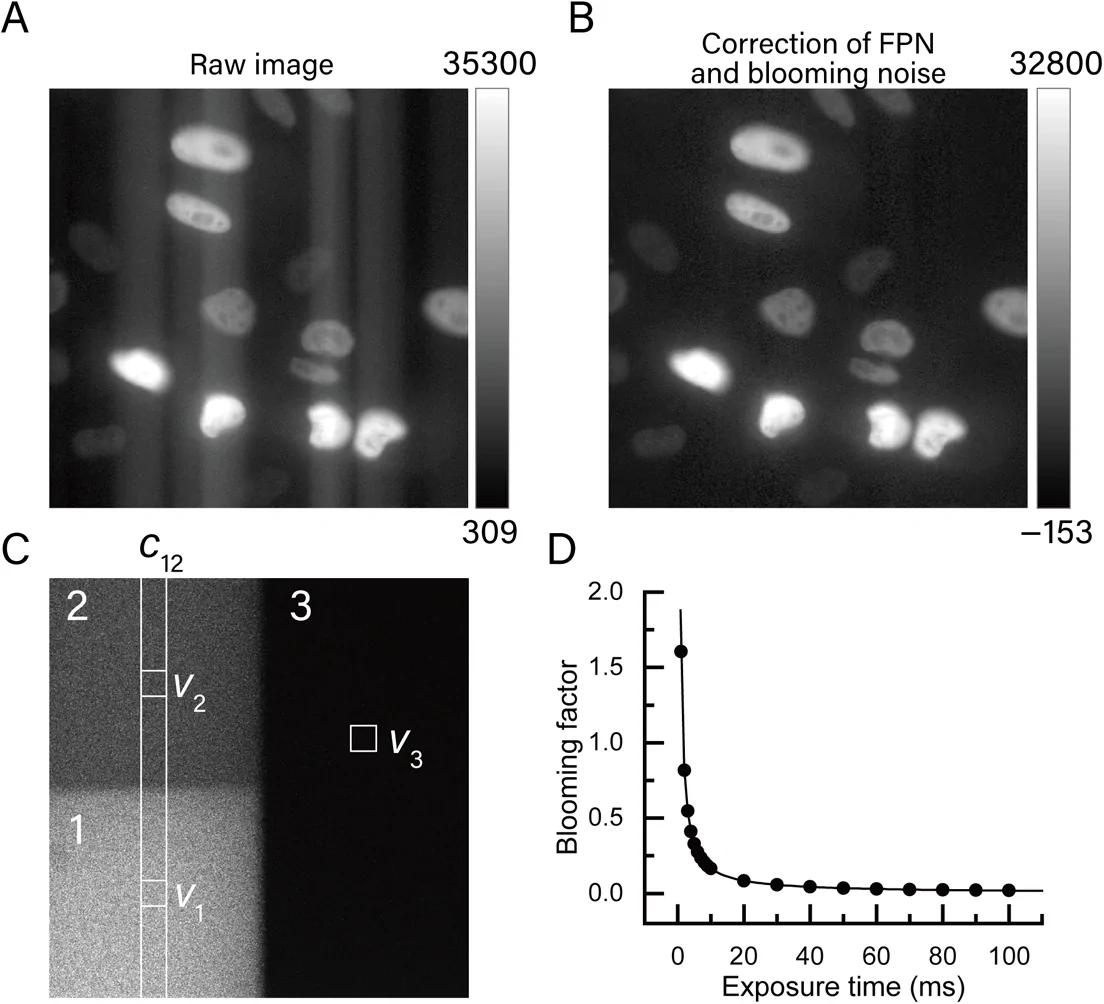
Blooming noise of observation using the EMCCD. (A) An example of blooming noise in a fluorescence image of HeLa cells expressing GeNL-H2B captured at an exposure time of 3 ms. (B) An example of blooming noise correction by Eq. (5). The image in (A) was corrected for FPN and blooming noise by Eq (5). (C) A test pattern projected on the EMCCD sensor for the measurement of blooming factor. A light was incident on region 1 and blooming noise was observed in region 2 but not in region 3. Average intensities in regions *v*_1_, *v*_2_, *v*_3_, and *c*_12_ were measured to calculate the blooming factor. (D) A plot of blooming factor as a function of exposure time. The plot was fitted with a function (blooming factor)=(2.121+0.002682 *t*_ex_)/(0.03551+1.2987 *t*_ex_), where *t*_ex_ is an exposure time.

**Figure 4:**
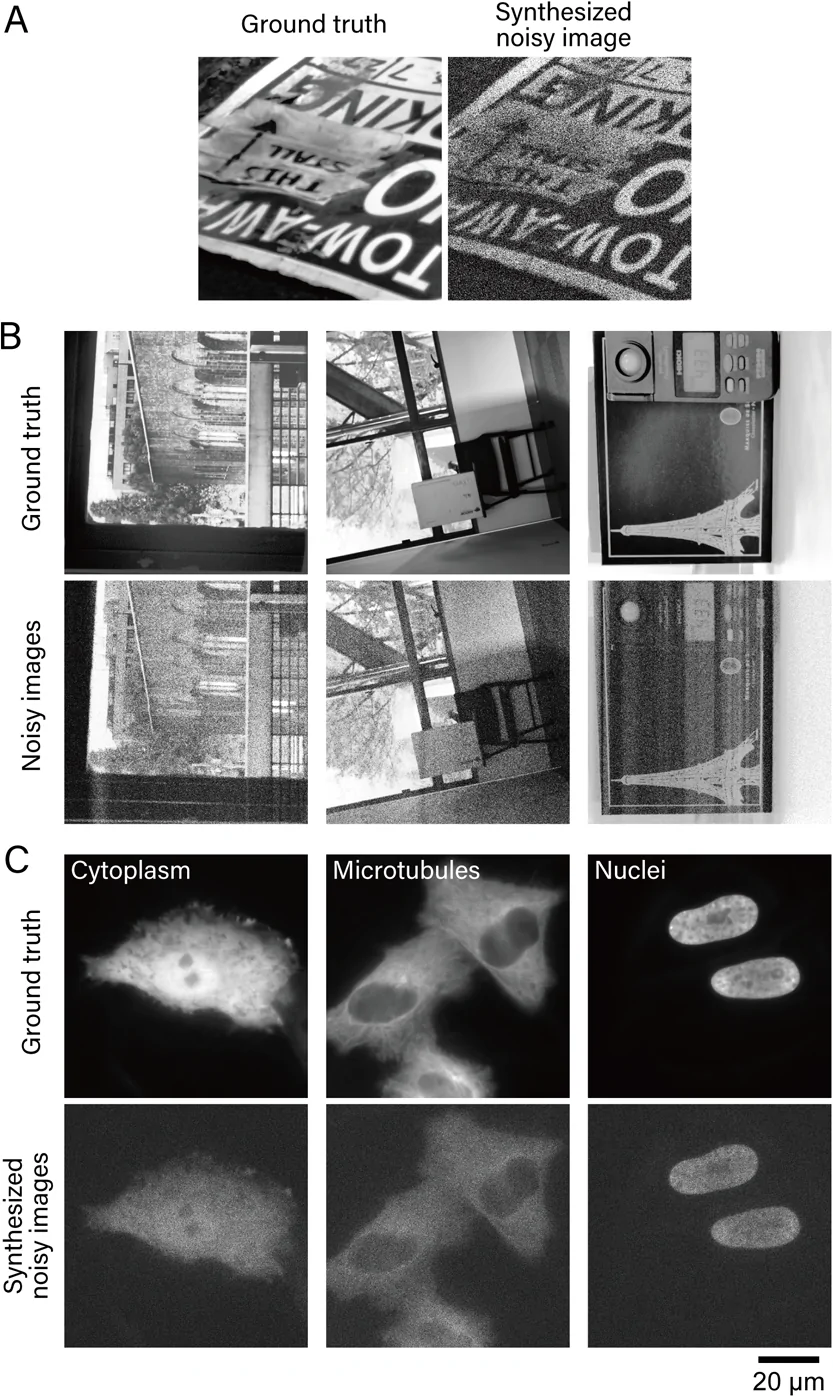
Examples of pairs of ground truth and synthesized noisy images. (A) A pair of ground truth and synthesized noisy images derived from SID dataset [17]. Adapted from ref [16]. (B) Pairs of ground truth and synthesized noisy images derived from images captured by the Andor iXon EMCCD. Adapted from ref [16]. (C) Pairs of ground truth and synthesized noisy images derived from microscopy images captured by the EMCCD. The noisy images were calculated by Eq. (8).

**Figure 5:**
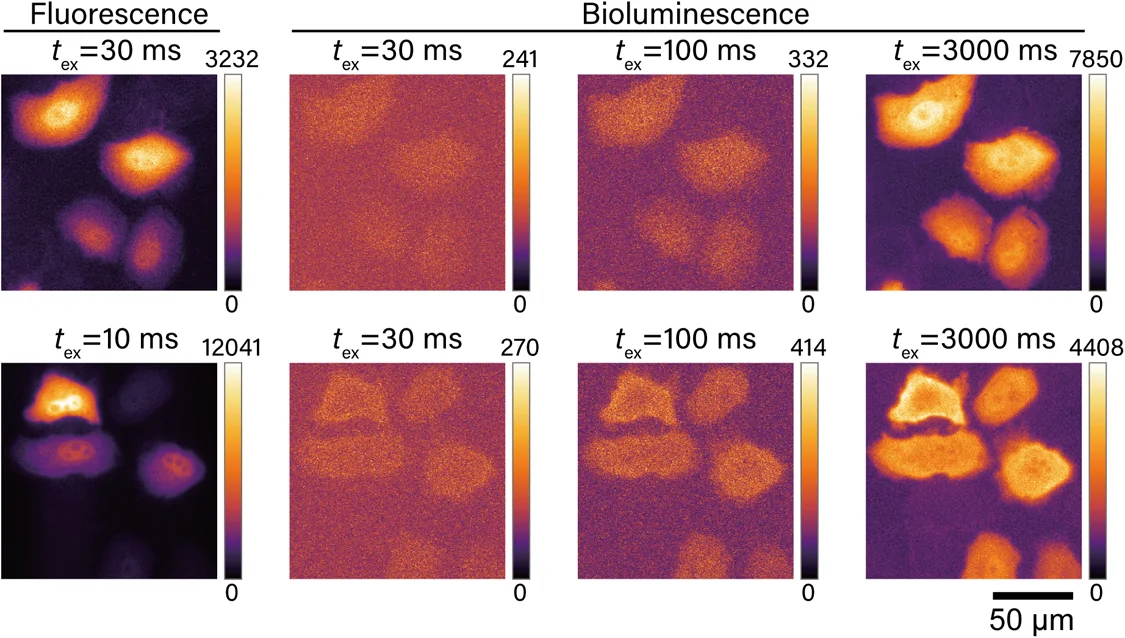
An example of bioluminescence and fluorescence images of GeNL expressed in cytoplasm of HeLa cells captured at various exposure times. FPN and blooming noises in the images were corrected by Eq. (5).

**Figure 6:**
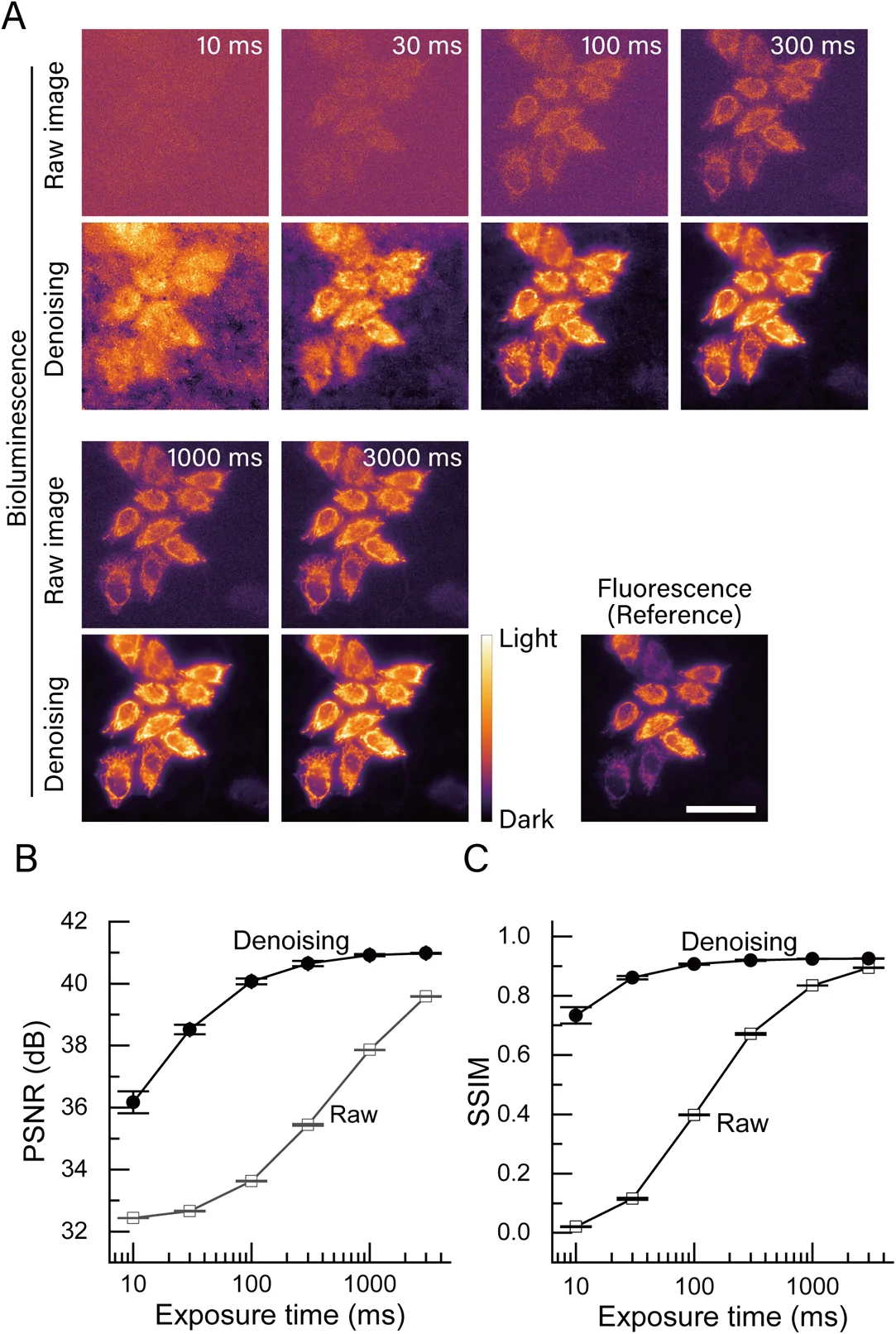
Denoising of microscopy images captured at various exposure times. (A) Raw and denoised images of bioluminescence of mitochondria labeled with GeNL expressed in HeLa cells and the results of denoising. The images are presented in pseudo-color scaled between intensities of 0.1% and 99.9% probabilities in the intensity distributions. The time values in the images are exposure times. A fluorescence image in the same field of view is also shown, and was used as a reference for the calculations of PSNR and SSIM. FPN and blooming noises in the images were corrected by Eq. (5). Scale bar, 50 μm. (B, C) Plots of PSNR and SSIM as a function of exposure time for raw and denoised images shown in panel (A).

**Figure 7:**
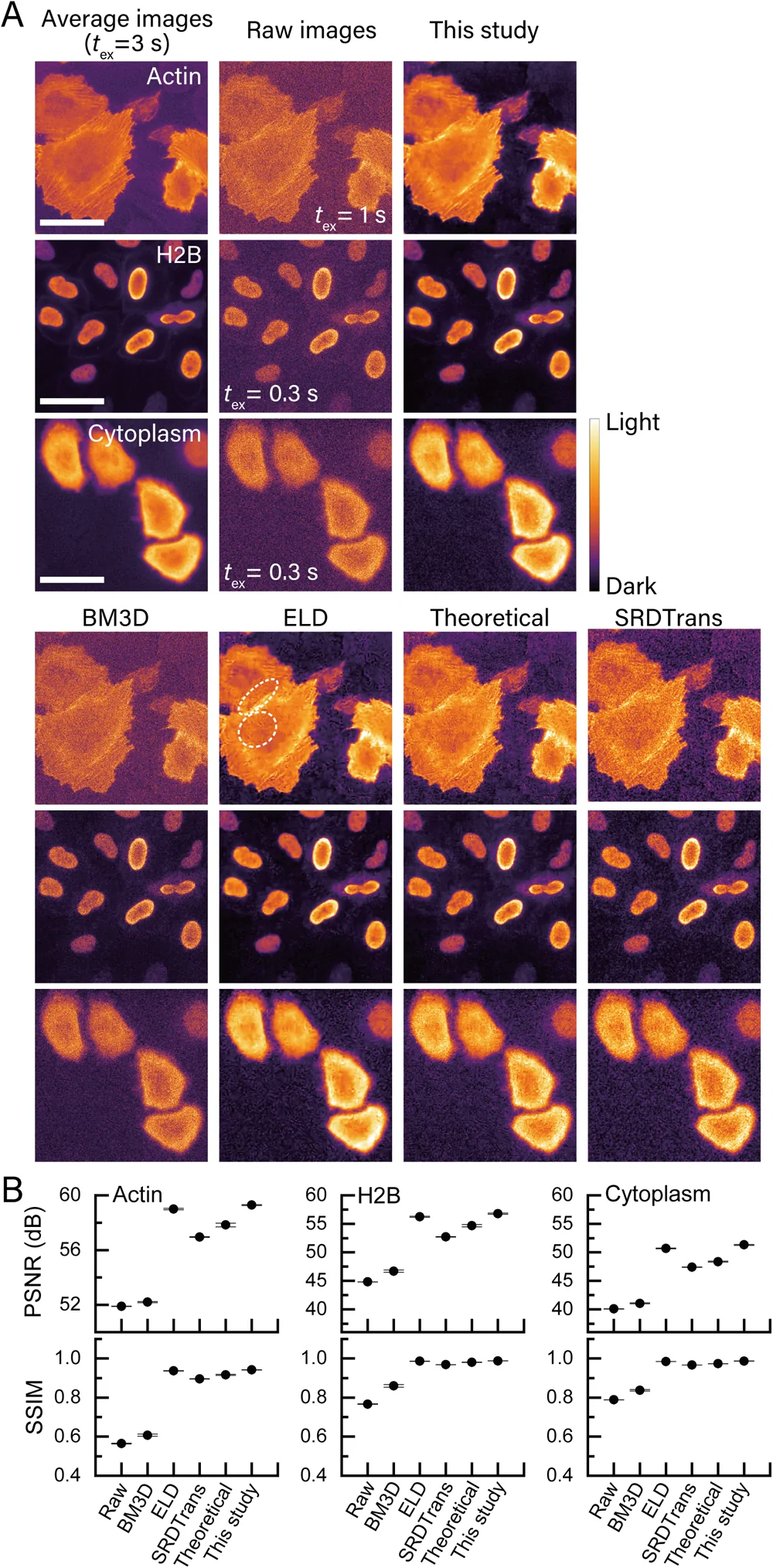
Comparison of denoising methods for bioluminescence images. (A) Bioluminescence images of actin, histone 2B (H2B), and cytoplasm labeled with GeNL expressed in HeLa cells and their results of denoising by this study’s method, BM3D, ELD, the theoretical noise model, and SRDTrans. Raw images captured at exposure times of 1 s for actin and 0.3 s for the others were corrected for FPN and then were applied to these denoising calculations. The average images in this panel are mean images of 4–5 frames captured at an exposure time of 3 s. In the denoised image of actin by ELD, unnatural patterns are enclosed by dashed circles. The average images were used as references for PSNR and SSIM analysis. The images are presented in pseudo-color scaled between intensities of 0.1% and 99.9% probabilities in the intensity distributions. Scale bars, 50 μm. (B) Plots of PSNR and SSIM for the denoising methods calculated from the images shown in panel (A).

**Figure 8:**
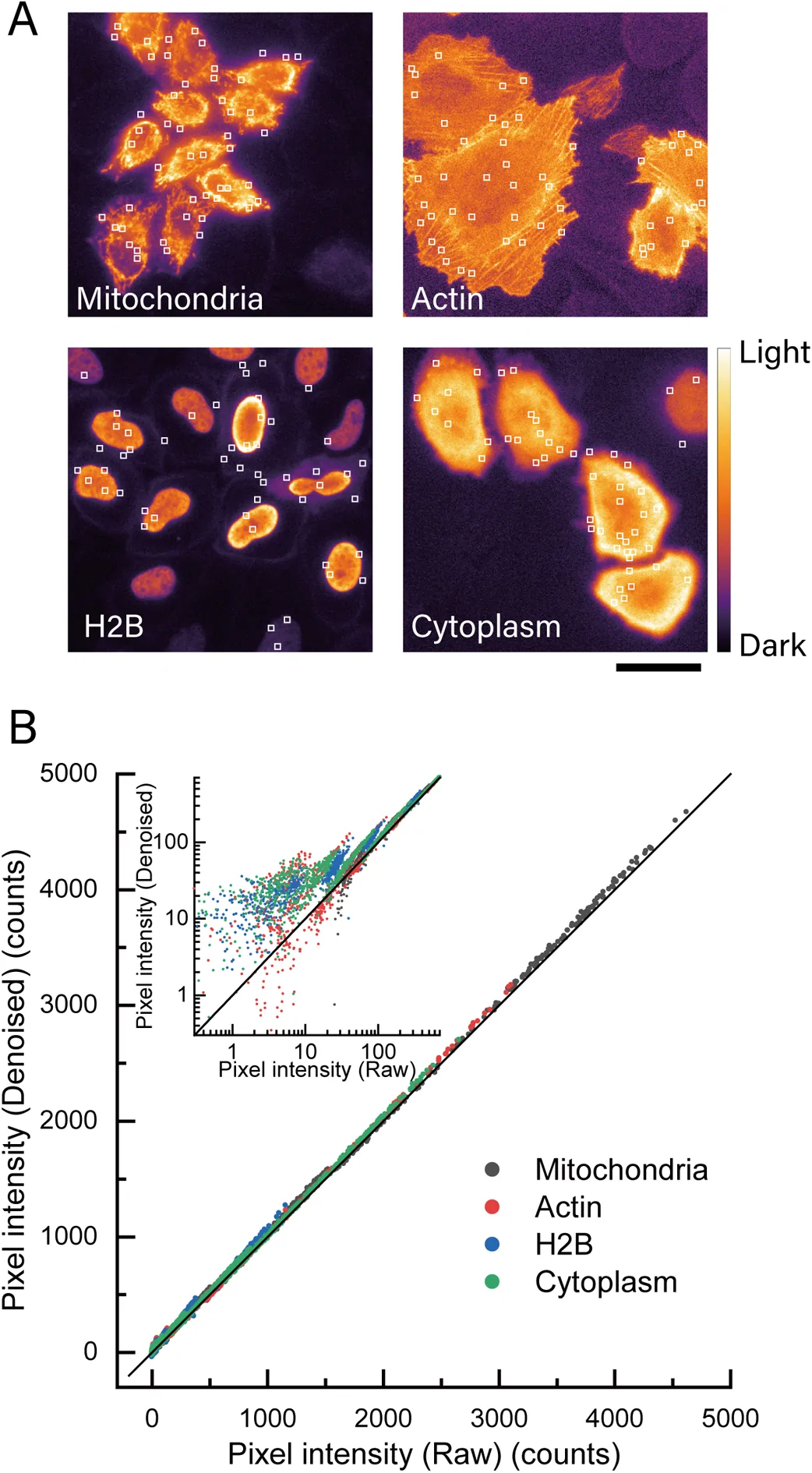
Fidelity of pixel intensity in denoised images. (A) Examples of ROIs generated for images of mitochondria, actin, histone 2B (H2B), and cytoplasm labeled with GeNL expressed in HeLa cells. Scale bar, 50 μm. (B) A plot of mean intensities of ROIs in denoised images versus mean intensities of the same ROIs in raw images. A diagonal line (*y*=*x*) is also shown in this panel. *R*^2^=0.998. The inset is a log-log plot of the data to show the low intensity range. The raw images used in this analysis were D_–bl,–FP_ (Eq. (5)).

**Table 1:** Summary of symbols in the noise model

Symbol	Description, emulation method	Units, values
**I**	Expectancy of the number of photonelectrons	photoelectrons
**N**_P_	Poisson shot noise. Random number from the Poisson distribution	photoelectrons
**N**_FP_	Fixed pattern noise.	counts
**N**_RO_	Readout noise. Random number from the normal distribution	counts
**N**_Q_	Quantization noise. Random number from the uniform distribution.	counts, –*q*/2≤(**N**_q_)*_i,j_*≤*q*/2^a^
*B*[*]	Blooming noise	Counts
*K*	Overall gain	counts/photoelectrons
*f*	Excess noise factor for **N**_P_. Random number from the uniform distribution	1.3≤*f*≤2^1/2^

^a^ *q* is a quantization step for analogue-to-digital conversion
